# Developing Digital Self-Regulation in Adolescence: A Developmental Self-Determination Model of Digital Self-Regulation

**DOI:** 10.1007/s10648-026-10183-y

**Published:** 2026-06-08

**Authors:** Ming-Te Wang

**Affiliations:** University of Chicago, W-16, WSSC 255, 969 E. 60th St., Chicago, IL 60637 USA

**Keywords:** Adolescent digital self-regulation, Digital literacy competence, Autonomy-supportive scaffolding, Parental mediation, School technology policy, Social media use

## Abstract

Digital technologies, such as social media, messaging, gaming, and emerging AI platforms, are deeply embedded in adolescents’ daily lives and shape their learning, social relationships, recreation, and identity development across contexts. In response to concerns about distraction, mental health, and academic engagement, schools and policymakers have increasingly emphasized restrictive approaches to digital use, including smartphone bans and access limitations. Although such policies may reduce short-term exposure, they often overlook adolescents’ developing self-regulatory capacities and basic psychological needs for autonomy, competence, and relatedness. Drawing on Self-Determination Theory and developmental science, this article introduces the Developmental Self-Determination Model of Digital Self-Regulation (DSMDS), a conceptual framework that reconceptualizes digital regulation as a developmental, capacity-building process rather than a problem of exposure control. Within DSMDS, digital literacy competence is defined as the knowledge and meaning-making foundation of digital self-regulation, whereas regulatory strategies are the behavioral and contextual tactics through which that understanding is enacted across devices, platforms, and activities. The model further theorizes how autonomy-supportive practices across peer, family, school, and policy contexts may scaffold regulation during periods of ongoing executive function development and how these supports may, under favorable conditions, be internalized over time. Importantly, DSMDS treats such progression as contingent rather than inevitable, emphasizing uneven literacy development, problematic engagement, and contextual pressures as important boundary conditions. The framework provides a developmentally grounded roadmap for research, policy, and educational practice aimed at fostering adolescents’ more intentional, context-sensitive, and self-directed engagement with digital technologies.

Social media and mobile technologies are now deeply embedded in adolescents’ daily lives, shaping how they learn, socialize, and construct their identities across school and out-of-school contexts (Nesi et al., 2018; Odgers & Jensen, 2020). In response to growing concerns about distraction, mental health, and academic engagement, schools and policymakers have increasingly turned to restrictive approaches, including school-wide smartphone bans and broader efforts to limit adolescents’ access to social media (Böttger & Zierer, 2024; Kates et al., 2018; United Nations Educational, Scientific and Cultural Organization (UNESCO), 2023). While these policies reflect legitimate concerns, they often rest on implicit assumptions that reducing access alone will improve outcomes. From a developmental and Self-Determination Theory (SDT) perspective (Deci & Ryan, 2000; Luna et al., 2015), however, such approaches risk overlooking adolescents’ fundamental psychological needs for autonomy, competence, and relatedness, as well as the ongoing development of executive function that constrains adolescents’ capacity for sustained self-regulation. When digital regulation is framed primarily as external control, it may reduce short-term exposure while doing less to support adolescents’ longer-term capacity to engage with digital technologies in intentional, self-directed, and developmentally adaptive ways, skills required for sustained attention, goal-directed use, and digital learning (Orben, 2020; Valkenburg et al., 2022).

This article introduces a developmentally informed, SDT-based framework for understanding and promoting adolescents’ digital self-regulation. I conceptualize digital self-regulation as a coordinated system in which digital literacy competence provides the knowledge and meaning-making foundation for the regulatory strategies adolescents use to manage engagement. Drawing on Self-Determination Theory and interdisciplinary developmental research, I propose a Developmental Self-Determination Model of Digital Self-Regulation (DSMDS) that integrates digital literacy competence with a typology of self-regulation strategies, specifies how parental and school practices may operate as complementary scaffolds, and generates hypotheses about how these supports may be internalized as adolescents’ executive function and motivation develop. By explicitly linking SDT, developmental neuroscience, and educational contexts, this framework offers a coherent roadmap for researchers, educators, and policymakers seeking to design digital regulation practices that support adolescents’ psychological needs while fostering durable self-regulatory skills.

## The Developmental Self-Determination Model of Digital Self-Regulation

### Model Overview

**Fig. 1:**
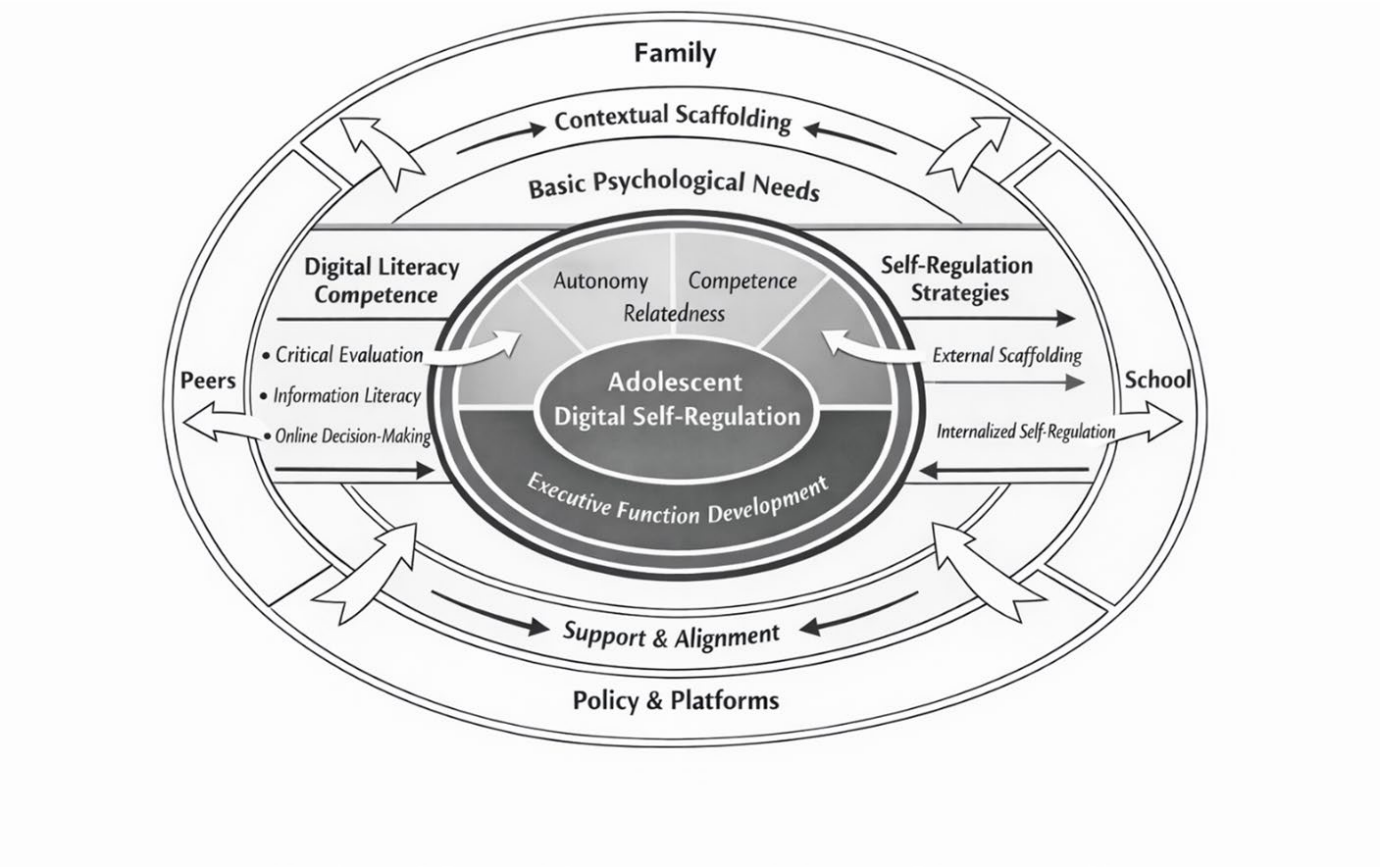
Developmental self-determination model of digital self-regulation

The Developmental Self-Determination Model of Digital Self-Regulation (DSMDS) explains how adolescents come to manage digital engagement in ways that are increasingly intentional, developmentally appropriate, and aligned with their goals, relationships, and well-being. Grounded in Self-Determination Theory and developmental science, the model conceptualizes digital self-regulation as a developmental process through which adolescents learn to navigate digital environments as their executive functions, motivational capacities, and social ecologies mature (Deci & Ryan, 2000; Luna et al., 2015; Ryan & Deci, 2000; Steinberg, 2008). In this framework (see Fig. 1 for a visual conceptualization), adolescents’ digital engagement is shaped by the joint operation of two core components—digital literacy competence and regulatory strategies—as well as by the autonomy-supportive or controlling conditions under which regulation occurs across family, school, peer, and policy contexts (Pérez-Verdugo and Barandiaran 2023; Stalmach et al. 2025a).

Digital literacy competence and regulatory strategies are distinct but interdependent components of the model. Digital literacy competence refers to the knowledge and meaning-making foundation of digital self-regulation: adolescents’ capacity to understand, interpret, evaluate, and reflect on digital environments, including the credibility of content, the social and algorithmic forces that shape online experience, and the likely emotional, behavioral, and relational consequences of digital use (Livingstone & Helsper, 2010; Martin, 2008; Remolina & Findlay, 2021). By contrast, regulatory strategies are the enactment layer: the behavioral and contextual tactics adolescents use to manage engagement in real time, such as planning use, restructuring environments, disabling notifications, setting time boundaries, or substituting alternative activities (Brevers & Turel, 2019; Duckworth et al., 2016; Flayelle et al., 2023). The model proposes that literacy may help adolescents recognize what requires regulation, why regulation matters, and which strategies may be appropriate, whereas the successful use of strategies is hypothesized to provide experiences that can strengthen perceived competence, reinforce internalization, and refine literacy over time (Zimmerman, 2000, 2002).

What develops over time, therefore, is not only adolescents’ ability to regulate technology use, but the relation between understanding and action. The model proposes that, under favorable developmental and contextual conditions, regulation may shift from more externally structured forms toward more self-endorsed and flexible forms of engagement as executive function matures and adolescents encounter more autonomy-supportive scaffolding from parents, schools, and other contexts (Grolnick et al., 2014; Reeve & Cheon, 2021; Soenens & Vansteenkiste, 2010). Importantly, DSMDS does not assume that this developmental progression occurs automatically or uniformly. Rather, drawing on SDT and developmental theory, DSMDS hypothesizes that internalization is more likely when adolescents are supported in ways that foster autonomy, competence, and relatedness while also reducing regulatory burden in high-demand digital environments (Ryan & Deci, 2017; West et al., 2025).

Within DSMDS, the target of regulation is adolescents’ digital engagement across devices, platforms, and activities rather than social media exposure alone. Because these targets differ in their demands and affordances, the model specifies that effective regulation may require different combinations of literacy, strategy use, and contextual scaffolding. In turn, the relevance of particular literacy demands, strategies, and supports is expected to vary depending on whether adolescents are managing access to devices, participation on specific platforms, or engagement in particular digital activities.

DSMDS is intended as a broad, domain-general developmental framework for understanding adolescents’ self-regulation across diverse forms of digital engagement, rather than as a model limited to social media distraction. At the same time, the model does not assume that all digital activities impose the same regulatory demands. Gaming, messaging, educational technologies, entertainment streaming, social media, and AI-based platforms may differ in their motivational structures, social affordances, reward contingencies, design features, and implications for autonomy, competence, and relatedness. Thus, DSMDS specifies a common developmental process—through which digital literacy, regulatory strategies, and contextual scaffolding may support more intentional and self-endorsed engagement—while also treating the specific targets, risks, and strategies of regulation as activity- and context-dependent.

I use digital technologies as an umbrella term that includes the devices through which adolescents connect (e.g., smartphones, tablets, laptops), the platforms and applications they use (e.g., social media, messaging, gaming, video-sharing, and educational platforms), and the specific activities they perform within those environments (e.g., scrolling, posting, messaging, searching, creating, learning, or consuming entertainment) (Giovanelli et al., 2020; Nesi et al., 2018; Odgers & Jensen, 2020). From this perspective, healthy digital engagement is not defined solely by access or duration, although time spent may still matter in some contexts (Orben, 2020). Duration is therefore treated as one relevant dimension of digital engagement rather than its sole indicator, becoming especially important when prolonged use becomes rigid, compulsive, or begins to interfere with adolescents’ own goals, valued relationships, responsibilities, or well-being. Rather, the model is concerned with whether digital engagement is self-endorsed, goal-aligned, context-sensitive, and flexible enough to support adolescents’ own values and developmental needs, including attention, learning, sleep, relationships, identity exploration, belonging, and well-being (Valkenburg et al., 2022). For example, digitally engaged behavior may be considered more developmentally adaptive when adolescents use technologies in ways that fit the demands of the setting and their broader goals, such as using messaging to maintain relationships without disrupting sleep, using digital tools to support learning without undermining attention during class, or disengaging from scrolling when use begins to conflict with academic, relational, or well-being priorities. From this perspective, “competent” and “meaningful” engagement are not treated as adult-imposed moral judgments, but as patterns of use that are increasingly intentional, context-sensitive, and supportive of adolescents’ functioning across key developmental domains.

Importantly, DSMDS treats adolescents’ own interpretations of digital engagement as central to evaluating whether use is meaningful or developmentally adaptive. For example, sustained engagement in digital art, fandom communities, gaming groups, or online friendships may be meaningful and developmentally adaptive when adolescents experience it as supporting self-expression, competence, identity exploration, or relatedness (Ito et al., 2019; Nesi et al., 2018; Parent, 2023), even if adults perceive the duration of use as excessive. From this perspective, the developmental question is not simply whether adults judge a pattern of use as appropriate, but whether adolescents can reflect on what the engagement provides, how it aligns with their values and goals, and whether it remains flexible enough to coexist with other developmental needs and responsibilities. Disagreements between adults and adolescents are therefore not treated as evidence that adolescents are necessarily misregulating; rather, they are understood as opportunities for autonomy-supportive dialogue, shared meaning-making, and reflection on both the benefits and tradeoffs of particular forms of digital engagement.

Taken together, this framing helps specify what DSMDS is designed to explain: how adolescents come to understand digital environments well enough to evaluate their demands, opportunities, benefits, and tradeoffs in relation to their own values and developmental needs, and how they translate that understanding into patterns of engagement that are increasingly self-endorsed, goal-aligned, context-sensitive, and developmentally adaptive.

I therefore turn first to digital literacy competence as the interpretive foundation of digital self-regulation, and then to the regulatory strategies through which that understanding is translated into action. 

### Digital Literacy Competencies as Need-Supportive Regulatory Capacities

Digital literacy competence is the interpretive and metacognitive foundation of digital self-regulation within DSMDS. It refers not merely to technical proficiency, but to adolescents’ capacity to understand digital environments, evaluate information and affordances, recognize social and algorithmic influences, and anticipate how digital engagement may affect their emotions, behavior, relationships, and longer-term goals (Livingstone & Helsper, 2010; Martin, 2008; Nguyen & Cheng, 2025; Remolina & Findlay, 2021). In SDT terms, these capacities are theorized to support adolescents’ needs for competence and autonomy by helping them feel informed, capable, agentic, and less reactive in digitally saturated environments (Deci & Ryan, 2000; Pérez-Verdugo & Barandiaran, 2023; Ryan & Deci, 2017). Digital literacy also has hypothesized motivational relevance: adolescents who better understand how platforms shape attention, mood, and interaction may be more likely to experience regulation as purposeful and self-endorsed rather than simply imposed from outside (Stalmach et al. 2025b). This interpretation is also compatible with qualitative evidence that students’ experiences of digital learning tools are closely tied to whether those tools support autonomy, competence, and, more unevenly, relatedness (Stalmach et al. 2025a). Within this framework, several interrelated literacy capacities are especially relevant for digital self-regulation.

One central literacy capacity is *critical evaluation*—the ability to assess the credibility, intent, and emotional impact of digital content. This capacity may support autonomy by helping adolescents make informed, self-endorsed choices rather than responding reactively to algorithmic cues or social pressures (Kahne & Bowyer, 2019; Nyhan & Reifler, 2010). This competency includes awareness of how platforms curate content and deploy persuasive design features (e.g., algorithmic amplification, notifications, streaks) to capture attention and shape behavior (Montag et al., 2019; Zuboff, 2019). Such algorithmic and design literacy may foster competence by increasing adolescents’ sense of mastery over complex media environments and enabling them to anticipate risks, recognize external influences on attention and emotion, and regulate exposure accordingly (Livingstone & Helsper, 2010; Remolina & Findlay, 2021). When embedded in dialogic contexts with parents, educators, or peers, critical evaluation may further support relatedness by legitimizing adolescents’ perspectives and encouraging shared meaning-making around digital experiences (Padilla-Walker et al., 2018; Soenens & Vansteenkiste, 2010).

*Intentional use*, defined as the capacity to align digital engagement with personally meaningful goals (e.g., learning, creativity, maintaining relationships), represents a more internalized form of regulation that directly reflects SDT’s emphasis on identified and integrated motivation (Deci & Ryan, 2000). Intentional use is expected to support autonomy by centering adolescents’ values and priorities, competence by reinforcing goal-setting and self-monitoring skills, and relatedness by promoting purposeful social connection rather than passive consumption (Martin, 2008; Zimmerman, 2000). Importantly, intentional use also involves adolescents’ capacity to articulate and communicate boundaries around availability and responsiveness (e.g., signaling when they are offline or prioritizing non-digital activities), positioning awareness of the need for boundaries as a relational literacy capacity and communicative self-advocacy as one way adolescents may enact that understanding in social contexts (Nesi et al., 2018; Weinstein & Przybylski, 2019). These capacities may emerge through the gradual internalization of externally scaffolded practices (e.g., time boundaries, environmental restructuring, or guided reflection), highlighting how digital literacy develops through coordinated self-, parent-, and school-level supports rather than through instruction alone (Padilla-Walker et al., 2018; Soenens et al., 2007).

Other literacy-relevant capacities include *metacognitive awareness of emotional responses* (e.g., recognizing when social comparison or exposure to certain content undermines well-being) and *recognition of when disengagement may be necessary* (e.g., stepping away from platforms during periods of stress or fatigue). These capacities further illustrate how digital literacy may support need-supportive regulation (Gross, 2015; Nesi & Prinstein, 2015). They may help adolescents anticipate when regulation is needed and protect psychological needs proactively rather than reactively, even in socially evaluative contexts (Baumeister et al., 2007; Hofmann et al., 2012). Together with awareness of the need for communicative boundary-setting, these capacities are theorized to support the shift from more externally regulated to more autonomously regulated media use, underscoring that digital literacy is not simply knowledge acquisition but a set of motivational, relational, and self-regulatory capacities that support healthy engagement across digital contexts (Martin, 2008; Mishra et al., 2015; Valkenburg et al., 2022).

Collectively, these digital literacy skills operate as an integrated system rather than as isolated competencies. Critical evaluation clarifies what features of digital environments warrant regulation, intentional use aligns engagement with personally meaningful goals, and metacognitive awareness helps adolescents recognize when regulation may be needed, particularly under conditions of emotional or social pressure. When combined, these competencies are expected to support adolescents’ needs for autonomy, competence, and relatedness by helping adolescents interpret digital engagement in ways that make more intentional and self-endorsed regulation possible.

### Development of Digital Literacy Competence

From a developmental perspective, digital literacy competence is not a unitary skill but a constellation of evolving capacities that mature alongside cognitive, social, and motivational development (Buckingham, 2007; Martínez-Bravo et al., 2022). In early adolescence, literacy may center on basic functional and social awareness, such as understanding platform norms, recognizing overt risks, and navigating peer interactions online. As adolescents’ abstract reasoning and perspective-taking abilities develop, literacy expands to include more sophisticated competencies, such as critically evaluating information credibility, recognizing algorithmic curation, and anticipating longer-term consequences of digital engagement (Best et al., 2011). These later-emerging competencies rely on advances in executive function, particularly cognitive flexibility and future-oriented thinking, and support adolescents’ growing sense of competence in complex digital environments (Diamond, 2013; Wartberg et al., 2021).

Equally important, digital literacy competence develops through experience and guided participation, not instruction alone (Ito et al., 2019; Rogoff, 2003). Adolescents refine literacy skills through repeated engagement with digital contexts, feedback from trusted adults and peers, and opportunities to reflect on successes and challenges. Drawing on broader developmental and motivational theory, autonomy-supportive contexts that invite adolescents to explain their choices, evaluate outcomes, and experiment with strategies are expected to be conducive to literacy development, as they support both competence and autonomy (Soenens & Vansteenkiste, 2010). Over time, literacy becomes increasingly integrated with adolescents’ identities and values (Moje & Luke, 2009), shaping not only how they interpret digital content but also how they define their goals for digital engagement. Within the DSMDS model, maturation of literacy is theorized to support movement from more externally guided regulation toward more self-directed, intentional, and value-consistent digital behavior, although DSMDS does not assume that this progression occurs fully, consistently, or for most adolescents (Ryan & Deci, 2000).

Further, digital literacy competence functions as a bridge between environmental demands and regulatory action. It equips adolescents to recognize how platforms shape attention and emotion, and to evaluate how digital engagement aligns with personal goals and values. However, literacy alone does not guarantee regulation (Demirbag & Bahcivan, 2021; Nigg, 2017). This limitation may be especially pronounced in digital environments that repeatedly reactivate attention through algorithmic recommendations, notifications, social feedback, and other habit-forming design features, making reflective choice more difficult to sustain even when adolescents understand the risks of continued engagement (Flayelle et al., 2023; Montag et al., 2019; Zuboff, 2019). Rather, DSMDS hypothesizes that literacy increases the likelihood that adolescents will deploy self-regulation strategies intentionally by clarifying what needs regulating, why regulation matters, and which strategies are likely to be effective in a given context. In other words, digital literacy may support the internalization of regulatory goals by translating external expectations (e.g., limits, norms) into personally meaningful reasons for action (Anthonysamy et al., 2020). These literacy processes inform adolescents’ selection and use of specific regulation strategies, shaping when they engage in activity-based substitution, how they deploy context- and technology-based constraints, and why they adopt anticipatory, time-structured approaches to managing digital use (Buchan et al., 2024; Hofmann et al., 2012). In this way, literacy may support the internalization of regulatory goals by helping adolescents interpret limits, norms, and prior experiences in more personally meaningful terms.

In sum, digital literacy competence may help adolescents recognize which aspects of digital environments require regulation, why regulation matters, and which responses may be most appropriate in a given context (Anthonysamy et al., 2020; Blanc et al., 2025). Whether that understanding is translated into action, however, depends on developmental capacity, motivation, and contextual supports (Demirbag & Bahcivan, 2021; Nigg, 2017). The next step in the model therefore concerns regulatory strategies: the enactment processes through which literacy-informed understanding is translated into action.

### Adolescents’ Digital Self-Regulation Approaches

In DSMDS, regulatory strategies are the context-sensitive behavioral and contextual tactics adolescents use to translate literacy-informed understanding into the management of everyday digital engagement. If digital literacy competence provides the knowledge and meaning-making foundation of digital self-regulation, regulatory strategies are the enactment layer through which that understanding is put into practice. These strategies include efforts to plan use, disengage from tempting or dysregulating content, restructure environments, establish time boundaries, and use technology-based tools to reduce distraction or support goals (Brevers & Turel, 2019; Duckworth et al., 2016; Flayelle et al., 2023). From this perspective, effective digital self-regulation depends not only on whether adolescents understand digital environments, but also on whether they can convert that understanding into action under real-world conditions shaped by immediate rewards, social demands, and competing goals. At present, the available literature suggests that some adolescents can enact digital regulation in specific ways, for example, by using notification controls, time boundaries, or environmental restructuring, but the evidence base remains limited with respect to whether adolescents can sustain such regulation consistently across settings and over time (Brevers & Turel, 2019). For this reason, DSMDS is intended less as a summary of a settled digital-specific evidence base than as a framework for organizing existing findings and specifying hypotheses about how sustained digital regulation may or may not develop. This evidentiary distinction is especially important in cases of problematic or addiction-like engagement, where adolescents may recognize the costs of use yet still struggle to disengage because high-reward cues, habit loops, and social reinforcement undermine the enactment of regulation.

This enactment process is shaped by the interaction of motivation, executive control, and contextual scaffolding. Drawing on SDT, DSMDS hypothesizes that adolescents are more likely to sustain regulation when strategies are aligned with identified or integrated goals, such as protecting sleep, maintaining academic focus, or preserving well-being, because such alignment makes regulation more self-endorsed and autonomy-supportive (Flayelle et al., 2023; Ryan & Deci, 2000). Successful strategy use also depends on executive function capacities such as inhibitory control, working memory, and planning, which remain under development across adolescence (Giordano et al., 2023; Reinecke et al., 2022; Zelazo & Carlson, 2012). As such, regulatory strategies often involve not only effortful self-direction but also external or self-imposed supports that reduce cognitive load and make regulation more feasible in digitally saturated environments. In DSMDS, then, digital self-regulation is understood not as willpower alone, but as a developmentally situated process through which adolescents coordinate internal motivation with behavioral tactics and contextual supports.

This enactment process is often complicated by affective reactivity, because adolescents frequently use social media to regulate emotions and needs for social belonging in real time (Reinecke et al., 2018; Weinstein et al., 2016). Even when adolescents endorse goals for limiting or redirecting digital engagement, enactment is shaped by affective processes as much as by executive control (Moreno & Uhls, 2019). Digital engagement often functions as a readily available way to manage boredom, stress, loneliness, and social uncertainty, particularly in contexts of heightened peer evaluation. From an SDT perspective, these affective drivers are closely linked to fluctuations in psychological need satisfaction and need frustration (Ryan & Deci, 2017; Vansteenkiste & Ryan, 2013): threats to relatedness (e.g., exclusion), competence (e.g., social comparison), or autonomy (e.g., feeling controlled) can intensify distress and increase the immediate appeal of digital engagement as a coping resource.

Consequently, I specify an affective pathway within digital self-regulation in which need frustration and stress reactivity increase the likelihood of compensatory or dysregulated engagement, especially when platforms provide immediate social feedback or distraction (Kardefelt-Winther, 2014). Affective arousal can also undermine the executive functions required for effortful regulation, helping explain why adolescents may return to social media despite awareness of costs and endorsed intentions to disengage (Arnsten, 2009; Diamond, 2013). This specification clarifies why effective scaffolding and strategy selection must address emotional triggers in addition to access and time: autonomy-supportive scaffolds that validate emotions and provide feasible routes to offline need satisfaction may reduce reliance on affect-driven coping, while strategies such as notification suppression, context shifts, and time-structured routines can reduce reactivity by lowering exposure to triggering cues and protecting goal-relevant contexts (e.g., sleep, learning, relationships; Soenens & Vansteenkiste, 2010).

Building on this interplay among motivational internalization, executive control, and affective reactivity, I identify an evidence-based typology of adolescents’ approaches to managing digital use (Brevers & Turel, 2019; Flayelle et al., 2023). These strategies represent distinct yet complementary regulatory pathways that differ in the extent to which they rely on adolescents’ executive effort versus external scaffolding (see Table 1). *Activity-based self-regulation* (e.g., engaging in alternative activities such as exercise, reading, shopping) reflects a more effortful form of regulation, drawing heavily on attentional control and autonomous motivation to redirect behavior toward intrinsically or instrumentally valued goals (Hofmann et al., 2012). In contrast, *context-based self-regulation strategies* operate by restructuring the environment to reduce exposure to digital cues, with access-eliminating contexts (e.g., leaving the phone in another room or in a no-service location) providing strong external scaffolds that minimize inhibitory demands, and access-limiting contexts (e.g., keeping the phone out of reach) requiring moderate executive control while preserving autonomy (Duckworth et al., 2016).

**Table 1:** Evidence-based typology of adolescent self-regulation strategies

Refined category label	SDT regulation type	Self-regulation mode	Developmental implication
Activity-based self-regulation	Autonomous / identified	Effortful	Higher self-control capacity
Context-based access management (Eliminating)	External → introjected	Highly scaffolded	Supportive for younger adolescents
Context-based access management (Limiting)	Identified	Moderately scaffolded	Transitional autonomy
Technology-supported self-regulation	External (self-chosen)	Scaffolded	Teachable skill
Time-structured self-regulation	Identified / integrated	Hybrid	Reflects planning maturity

*Technology-supported self-regulation strategies* (e.g., disabling notifications, using Do Not Disturb) further externalize regulation by embedding constraints within the device itself, effectively offloading executive demands while allowing adolescents to retain agentic choice over when and how such tools are deployed (Brevers & Turel, 2019). Finally, *time-structured self-regulation strategies* (e.g., setting specific windows for digital engagement, gaming, messaging, or social media use, or using app timers) reflect anticipatory self-regulation, integrating planning and rule-based control with motivational alignment around sleep, academic, or well-being goals (Flayelle et al., 2023). Taken together, this typology underscores that digital self-regulation is enacted through multiple strategies whose usefulness may vary across developmental stages and digital contexts.

DSMDS further proposes that digital self-regulation is more likely to become internalized when adolescents experience regulation as self-endorsed, meaningful, and developmentally appropriate. Because executive function remains under development across adolescence, especially in contexts saturated with immediate rewards and social evaluation, external scaffolding from parents, schools, peers, policy, and platform design may reduce regulatory burden while supporting growing autonomy (Luna et al., 2015; Steinberg, 2008; West et al., 2025). Importantly, such scaffolds are not merely temporary supports to be discarded once self-regulation improves; they may remain developmentally useful when they help adolescents and emerging adults manage digital environments designed to sustain attention, encourage re-engagement, and make disengagement difficult. In this framework, scaffolding is not treated as the opposite of autonomy. When it is predictable, explanatory, and developmentally calibrated, it may help adolescents experience success, competence, and shared purpose, thereby potentially supporting the longer-term internalization of regulatory practices (Grolnick et al., 2014; Reeve & Cheon, 2021; Soenens & Vansteenkiste, 2010). This developmental logic is especially important during adolescence, when neurocognitive systems involved in executive control are still maturing.

### Developmental and Neuroscience Foundations of Digital Self-Regulation

From a developmental perspective, adolescence represents a period in which digital self-regulation is likely to be both especially important and especially difficult (Przybylski et al., 2013; Siebers et al., 2022). Neurodevelopmental research indicates that executive function capacities, including inhibitory control, cognitive flexibility, and future-oriented planning, continue to mature into late adolescence and early adulthood, reflecting ongoing development of prefrontal systems and their coordination with affective and reward-related processes (Diamond, 2013; Luna et al., 2015; Steinberg, 2008). At the same time, adolescents are highly sensitive to immediate rewards, salient social cues, and peer evaluation, all of which are prominent features of contemporary digital environments (Nesi et al., 2018; Weinstein & Przybylski, 2019). These broader developmental patterns suggest that expectations for fully autonomous digital self-regulation during adolescence may often be developmentally misaligned. In DSMDS, this developmental context helps explain why external scaffolds, structured supports, and developmentally calibrated participation in regulatory decisions may be particularly relevant during adolescence and may remain useful as adolescents move into early adulthood.

Within this framework, DSMDS proposes that adolescents’ digital regulatory strategies may be understood not as a simple progression from external scaffolding to independent self-regulation, but as a developing coordination between internal regulatory capacities and external supports. Strategies that reduce cognitive load (e.g., environmental restructuring, technology-based supports, device-free routines, and clear temporal boundaries) may be particularly useful when executive control is still maturing, but their relevance does not necessarily disappear in late adolescence or adulthood (Brevers & Turel, 2019; Duckworth et al., 2016; Flayelle et al., 2023; Reinecke et al., 2022). Because many digital platforms are intentionally designed to sustain engagement through social feedback, algorithmic reinforcement, persistent notifications, and other persuasive features, even highly motivated and digitally literate individuals may benefit from ongoing environmental and design-level supports (Flayelle et al., 2023; Montag et al., 2019; Zuboff, 2019).

DSMDS therefore treats development as involving the increasing capacity to select, interpret, negotiate, and use supports in self-endorsed ways, rather than as the gradual elimination of external supports. Younger adolescents may often require more adult-structured limits or access-reducing strategies, whereas older adolescents and emerging adults may increasingly participate in choosing, adapting, and maintaining supports that fit their goals, contexts, and vulnerabilities. In this sense, internalization does not mean that regulation becomes purely independent. Rather, it means that adolescents become better able to understand why supports are useful, align them with personally meaningful goals, and use them flexibly across changing digital environments. Framed this way, DSMDS specifies how internal capacities and external scaffolds can remain dynamically coordinated across adolescence, the transition to adulthood, and potentially beyond.

This coordination framework remains a conceptual proposition intended to organize future research rather than a settled empirical sequence in the digital-specific literature. Drawing on developmental theory and broader self-regulation research, DSMDS specifies that younger adolescents, whose executive control and anticipatory planning are still emerging, may often benefit from strategies that substantially reduce exposure to digital temptations or offload regulatory demands, including access-reducing routines, environmental restructuring, and technology-supported constraints (Best et al., 2011; Duckworth et al., 2019). As adolescents mature and demonstrate greater self-awareness, planning capacity, and value-based motivation, some may become better positioned to participate in selecting and adapting more anticipatory strategies, such as time-structured planning or activity-based substitution, especially when supports are paired with structure, explanation, and opportunities for shared decision-making (Padilla-Walker et al., 2012; Reeve & Cheon, 2021; Vygotsky, 1978). However, whether, when, and for whom such coordination becomes more self-endorsed is likely to depend on multiple conditions, including digital literacy, contextual scaffolding, affective reactivity, peer pressures, and the design features of digital environments themselves. Thus, the developmental contribution of DSMDS is not to claim that adolescents reliably move away from external supports, but to provide a theoretically grounded framework for age- and context-sensitive hypotheses, intervention design, and future empirical testing.

### Boundary Conditions and Challenges to Internalization

Although DSMDS outlines a developmental logic through which digital literacy, regulatory strategies, and contextual scaffolding may support more self-endorsed digital regulation over time, the model does not assume that this process unfolds smoothly or successfully for all adolescents. Several boundary conditions may disrupt or constrain internalization. First, digital literacy may remain weak or unevenly developed, limiting adolescents’ ability to accurately evaluate digital content, recognize persuasive design features, or anticipate the longer-term consequences of their engagement (Livingstone & Helsper, 2010; Martínez-Bravo et al., 2022). In such cases, adolescents may lack the interpretive foundation needed to identify what requires regulation in the first place. More broadly, DSMDS does not posit that most adolescents will develop highly elaborated digital literacy or consistently translate such literacy into autonomous regulation. For many youth, movement away from externally guided regulation may remain partial, fragile, or infrequent, particularly when literacy is weak, scaffolding is inconsistent, or digital environments place heavy demands on attention, emotion regulation, and social belonging. Importantly, this challenge is not limited to adolescents; many adults also struggle to critically evaluate digital environments and persuasive design features, which underscores that digital literacy cannot be assumed even among those providing guidance or scaffolding. Available descriptive evidence further suggests that digital literacy develops unevenly rather than robustly for many adolescents. In a UNICEF multi-country study of adolescents’ online engagement in East Asia and the Pacific, young people were described as digitally savvy and heavily online, yet they largely used platforms for entertainment and leisure rather than for knowledge acquisition or skill development, and the report concluded that these patterns were not necessarily translating into stronger digital literacy or critical thinking (United Nations Children’s Fund, 2021).

Second, even when adolescents understand digital risks and recognize the value of regulation, they may still struggle to regulate effectively in practice. Population-level evidence also suggests that regulation failures are not rare. In the 2021/2022 Health Behaviour in School-aged Children survey across Europe, central Asia, and Canada, 11% of adolescents were classified as showing problematic social media use, up from 7% in 2018, 12% were at risk of problematic gaming, and 36% reported being in constant online contact with friends and others (Boniel-Nissim et al., 2024; World Health Organization, 2024). Knowledge alone does not eliminate competing motives, limited executive capacity, or situational temptations, particularly in environments designed to capture attention and reward rapid re-engagement (Demirbag & Bahcivan, 2021; Nigg, 2017).

Addiction-like or problematic digital engagement further complicates the relation between literacy and regulation. In such cases, adolescents may retain some awareness of persuasive design features, overuse risks, or personal goals, yet still struggle to disengage because platform design can repeatedly reactivate attention, social motivation, and habit-based responding. Features such as algorithmic recommendations, infinite scroll, autoplay, streaks, social feedback metrics, notifications, and variable reward schedules may create cyclical engagement loops in which each interaction generates new cues for continued use or rapid return (Boniel-Nissim et al., 2024; Flayelle et al., 2023; Montag et al., 2019; World Health Organization, 2024; Zuboff, 2019). From the DSMDS perspective, problematic use therefore does not simply indicate weak digital literacy or insufficient motivation. Rather, it may reflect a mismatch between adolescents’ developing regulatory capacities and digital environments designed to intensify reward, reduce stopping cues, and make disengagement effortful. Under these conditions, literacy and self-regulatory intentions may be outcompeted by cue-driven habits, social reinforcement, and platform-level design pressures, disrupting both the enactment of regulation and the internalization process itself. This makes external scaffolding, environmental restructuring, technology-supported constraints, and platform- or policy-level design changes especially important rather than optional.

Third, affective reactivity may override intentions when adolescents turn to digital engagement to cope with boredom, loneliness, stress, or social uncertainty, making regulation especially difficult in moments of heightened need frustration or emotional arousal (Kardefelt-Winther, 2014; Moreno & Uhls, 2019; Ryan & Deci, 2017). Finally, peer and contextual pressures may make self-endorsed regulation socially costly, especially when constant availability, visibility, or responsiveness are tied to belonging and status (Beyens et al., 2016; Nesi et al., 2018; Parent, 2023). These boundary conditions underscore that digital self-regulation should not be treated as an inevitable developmental achievement, but as a context-sensitive process that may require sustained support, adaptive scaffolding, and environments that reduce rather than intensify regulatory demands.

### Parental Digital Regulation and Mediation Approaches

From the DSMDS perspective, parental strategies to monitor, guide, and limit adolescents’ digital engagement can be understood as forms of external regulatory scaffolding that shape youths’ opportunities for autonomy, internalization, and skill development. Because adolescents’ executive function and self-regulatory capacities remain under development, parents often play an important role in structuring the digital environment through oversight, communication, rules, and support (Buijzen & Valkenburg, 2005; Livingstone & Helsper, 2008). However, DSMDS does not assume that parental mediation is uniformly beneficial or harmful in itself. Rather, drawing on SDT, the model specifies that the developmental implications of parental mediation depend on how it is enacted, including whether it supports adolescents’ autonomy and competence, whether it is experienced as legitimate and predictable, and whether it is calibrated to adolescents’ developmental readiness and relational context (Bradshaw et al., 2025; Ryan & Deci, 2017; Soenens & Vansteenkiste, 2010; Wisniewski et al., 2017). Viewed through this lens, parental mediation strategies can be interpreted as differing in the extent to which they function as collaborative, competence-supporting scaffolds versus controlling constraints, with corresponding implications for whether adolescents are likely to internalize digital self-regulation over time. Table 2 presents a parallel typology of adolescent self-regulation and parental mediation strategies.

**Table 2:** Parallel typology of adolescent self-regulation and parental mediation strategies

Regulatory function	Adolescent strategy	Parent strategy	SDT characterization	Executive function role
Motivational & cognitive guidance	Activity-based self-regulation (doing alternative activities)	Active mediation (discussion, explanation, guidance)	Autonomy-supportive; promotes identified/integrated regulation	Builds planning, decision-making, attentional control
Contextual access control (Strong)	Access-eliminating contexts (no phone, no Wi-Fi)	Restrictive mediation (platform bans, strict limits)	External regulation → potential internalization if justified	Minimizes inhibitory demands
Contextual access control (Moderate)	Access-limiting contexts (phone out of reach)	Non-intrusive inspection (knowing accounts, friending)	Structured autonomy support	Moderately reduces temptation while preserving agency
Embedded regulatory tools	Technology-supported self-regulation (DND, notifications off)	Restrictive mediation (time limits, activity restrictions)	Scaffolded regulation	Offloads executive control
Temporal boundary setting	Time-structured self-regulation (timers, schedules)	Restrictive mediation (time rules)	Identified regulation when goal-aligned	Supports planning and rule adherence
Direct oversight & enforcement	NA	Authoritarian surveillance (passwords, message checks)	Controlling external regulation	Bypasses adolescent EF development

#### Active Mediation as Collaborative Motivational Scaffolding

Active mediation strategies—such as discussing appropriate information disclosure, explaining social media risks, and encouraging disengagement from uncomfortable interactions—closely align with autonomy-supportive socialization in SDT (Ryan & Deci, 2017; Soenens & Vansteenkiste, 2010). These practices do not directly restrict access or behavior; instead, they target adolescents’ cognitive and motivational processes, fostering understanding, reflection, and value alignment (Clark, 2011; Nathanson, 2001). In parallel to adolescents’ *activity-based* and *time-structured* self-regulation strategies, active mediation may support the internalization of regulatory goals by strengthening adolescents’ competence (knowing how to navigate risks) and autonomy (choosing to regulate behavior based on understanding rather than fear of punishment). From an executive function standpoint, active mediation may help adolescents develop anticipatory control and decision-making skills that can later be enacted independently in digital contexts (Steinfeld, 2021). For example, a parent might collaboratively establish phone-free homework windows, explain that the goal is to protect attention and reduce distraction, and then gradually involve the adolescent in adapting and maintaining those boundaries as self-regulatory competence develops.

#### Restrictive Mediation as Contextual and Temporal Scaffolding.

Restrictive mediation strategies—such as limiting access to particular platforms, activities, apps, or time-intensive forms of digital engagement—function as context-based and temporal scaffolds that closely parallel adolescents’ own access-eliminating, access-limiting, and time-structured strategies. These parental practices reduce exposure to high-risk or highly tempting digital environments, thereby lowering the inhibitory and attentional demands placed on adolescents’ developing executive function (Gkora & Drigas, 2024; Hofmann et al., 2012). Within the DSMDS model, restrictive mediation is hypothesized to support self-regulation when it is clearly justified and developmentally appropriate, helping adolescents experience successful regulation and potentially internalize boundaries. However, when restrictions are perceived as arbitrary or overly controlling, they are more likely to remain externally regulated and less likely to promote long-term self-regulatory competence.

#### Authoritarian Surveillance as Controlling External Regulation

Authoritarian surveillance strategies—such as requiring access to passwords or actively monitoring private messages and interactions—represent a form of highly controlling external regulation. These practices rely on direct oversight rather than adolescents’ own executive or motivational capacities and closely resemble access-eliminating strategies imposed by authority rather than chosen by the adolescent. From the DSMDS perspective, such strategies are likely to thwart autonomy and may undermine trust, potentially limiting internalization of self-regulatory goals. Although surveillance may reduce certain risks in the short term, especially for younger adolescents or those facing acute safety concerns, it provides limited opportunities for adolescents to practice independent regulation and may weaken the development of competence-based self-regulation over time.

#### Non-Intrusive Inspection as Transitional Monitoring

Non-intrusive inspection strategies—such as knowing adolescents’ accounts, viewing public profiles, or being connected as a social media “contact”—occupy a middle ground between autonomy support and control. These strategies parallel adolescents’ access-limiting self-regulation practices by increasing accountability and visibility without fully eliminating privacy or agency (Racz & McMahon, 2011). When framed transparently and used collaboratively, non-intrusive inspection can function as a moderate scaffold, signaling parental presence while still allowing adolescents to exercise discretion and executive control (Coyne et al., 2014; Livingstone & Helsper, 2008). This approach may be particularly well suited for supporting adolescents during transitional developmental periods, as it balances safety monitoring with opportunities for internalization.

### Integrated Parent–Child Regulatory System

Adolescents’ self-regulation strategies and parents’ mediation practices can be understood as parts of an interdependent regulatory system rather than as separate or competing influences. Parental strategies help structure the contexts in which adolescents learn to manage digital engagement, while adolescents’ own regulatory efforts shape how parental mediation is interpreted, negotiated, and adjusted over time (Beyens et al., 2022; Ho et al., 2020). Within DSMDS, digital literacy competence remains the knowledge and meaning-making foundation of digital self-regulation, whereas regulatory strategies are the enactment layer through which that understanding is translated into action. Parental mediation enters this system as a contextual scaffold that may strengthen, constrain, or redirect adolescents’ opportunities to practice that translation, depending on how mediation is framed, timed, and relationally experienced.

DSMDS further proposes that parental digital mediation may shift over time from more direct forms of control toward more collaborative forms of scaffolding, although this should be understood as a developmental expectation rather than a universal sequence. Early in adolescence, when executive function, judgment, and anticipatory planning are still emerging, parents may be more likely to rely on restrictive mediation or closer monitoring to reduce exposure to high-risk or highly tempting digital contexts (Livingstone & Helsper, 2008; Steinberg, 2008). As adolescents demonstrate greater competence, responsibility, and self-awareness, parents may become better positioned to rely more on non-intrusive inspection, active mediation, and collaborative rule-setting, approaches that preserve greater room for adolescent agency while still providing structure and guidance (Grolnick et al., 2014; Padilla-Walker et al., 2012; Vygotsky, 1978). Whether such shifts are likely to support internalization is a theoretical and empirical question that likely depends on timing, relational trust, developmental readiness, and the extent to which parental practices are experienced as legitimate and explanatory rather than controlling (Grusec & Goodnow, 1994; Kerr et al., 2012; Smetana, 2017).

Timing is a critical but often under-theorized component of parental mediation. The same strategy may have different implications depending on when it is deployed in adolescence. For example, introducing autonomy-supportive strategies too late after prolonged periods of strict control may be experienced as abrupt or inconsistent, undermining trust (Collins & Laursen, 2004; Feldman & Quatman, 1988). Conversely, withdrawing scaffolds too early, before adolescents have developed sufficient executive capacity, may increase risk exposure and regulatory failure (Steinberg, 2014). From the DSMDS perspective, optimal timing involves gradually increasing adolescents’ decision-making authority in tandem with their demonstrated competence, ensuring that autonomy is experienced as supported rather than abandoned. This highlights the importance of viewing parental mediation as developmentally contingent, rather than uniformly beneficial or harmful (Smetana, 2017).

Responsiveness to adolescents’ developmental signals (e.g., growing self-awareness, expressed values, or responsible behavior) is central to adaptive parent–adolescent regulation (Smetana, 2017). Responsive parents adjust their strategies based on adolescents’ feedback, struggles, and successes, signaling respect for adolescents’ emerging agency (Kerr et al., 2012). This responsiveness may foster a feedback loop in which adolescents’ self-regulatory efforts are recognized and reinforced, potentially promoting further internalization. In contrast, unresponsive or rigid mediation strategies may generate negative cascades, including secrecy, resistance, or disengagement from parental guidance (Grusec & Goodnow, 1994). These reciprocal processes underscore that parental mediation does not merely constrain behavior in the moment but shapes adolescents’ long-term motivational orientation toward regulation, authority, and self-governance (Tilton-Weaver et al., 2014).

### School as a Developmental Context for Adolescent Digital Self-Regulation

School contexts are a major developmental setting in which adolescents’ digital self-regulation is structured, interpreted, and practiced. From the DSMDS perspective, schools can be understood as developmental regulatory environments that structure when, where, and how digital engagement is permitted, interpreted, and practiced (Darling-Hammond et al., 2020; Eccles & Roeser, 2011; Wang & Degol, 2016; Wang et al., 2020). Unlike families, schools regulate digital behavior through institutional norms, classroom routines, instructional practices, and formal policies, positioning them as important contexts for both external scaffolding and socialization. Importantly, DSMDS does not treat school-based regulation as inherently supportive or controlling. Instead, the model proposes that the developmental value of school practices depends on how they are designed and enacted, including whether they provide structure with clear rationale, preserve opportunities for agency, and help students build the literacy and self-regulatory capacities needed for increasingly self-directed digital engagement (Reeve, 2012; Reeve & Tseng, 2011; Wang et al., 2019).

DSMDS further proposes that schools and districts may support adolescents’ digital self-regulation by designing policies and practices that reduce regulatory burden while gradually creating space for agency, reflection, and skill development. This should be understood as a developmentally informed design principle rather than a universally established sequence. At the universal level, schools may provide structure through clear technology-use expectations, predictable routines, and developmentally calibrated boundaries that help protect attention, learning, and well-being (Assor et al., 2002; Eccles & Roeser, 2011; Meyer et al., 2019). However, the model suggests that such structures are hypothesized to be more likely to support internalization when they are paired with explanation, consistency, and opportunities for students to understand why regulation matters, rather than when they operate only as punitive restrictions. In this sense, DSMDS proposes that school-based regulation may be most developmentally useful not when it merely limits access, but when it combines external structure with opportunities to practice literacy, self-monitoring, and increasingly self-endorsed forms of digital regulation (Hobbs, 2017; Jones & Mitchell, 2016; Livingstone et al., 2021).

At the school level, developmentally supportive practices can be embedded into daily routines and instructional design. Structured device-use norms during instructional periods can be paired with intentional “choice windows” that allow students to decide when and how technology is used for academic or creative purposes. These practices parallel parental scaffolding strategies by providing predictable structure and, where appropriate, increasing flexibility as students demonstrate regulatory competence (Patall et al., 2008; Reeve, 2012). When educators communicate expectations in a non-controlling manner, acknowledging students’ perspectives and emotions, schools reinforce adolescents’ sense of relatedness and trust, which is essential for internalization (Reeve & Tseng, 2011).

Schools can further support adolescents’ developmental needs by explicitly cultivating competence in digital self-regulation through instruction and guided practice (Chiu et al., 2022; Jones & Mitchell, 2016). Integrating digital and media literacy, self-management skills, and collaborative learning structures into curricula allows students to learn how to evaluate online content, manage attention, set personal boundaries, and practice digital regulation in socially supported ways (D’Elia et al., 2025; Hobbs, 2017; Nguyen & Oanh, 2025). For example, schools might pair structured classroom device norms with explicit instruction in attention management, critical evaluation of online content, guided reflection on when digital tools support versus undermine learning, and cooperative learning routines that distribute responsibility and reinforce engagement through shared academic goals (D’Elia et al., 2025; Nguyen & Oanh, 2025). Instructional approaches that involve reflection, goal setting, and self-monitoring are hypothesized to promote internalization by enabling students to experience mastery and success in regulating their own behavior (Zimmerman, 2002). When students are invited to participate in shaping classroom norms or reflecting on their digital habits, schools reinforce autonomy and competence simultaneously, positioning self-regulation as a skill to be developed rather than a rule to be obeyed (Mitra, 2004).

Finally, schools play a critical role in supporting adolescents’ need for relatedness by shaping peer norms around digital engagement. Because much social media use is motivated by peer connection, school-based approaches that encourage collaborative learning, face-to-face interaction, and respectful digital citizenship can reduce overreliance on online platforms for belonging (Ribble & Park, 2019). Peer-led initiatives, advisory programs, and restorative practices can foster shared responsibility for digital well-being, aligning peer norms with self-regulatory goals. At the district level, policies that allow flexibility across developmental stages, while maintaining coherence with family and community expectations, may create conditions under which adolescents increasingly participate in regulating their digital behavior (Eccles & Roeser, 2011).

### Integrated School-Parent–Child Regulatory System

Schools can also be understood as a meso-level regulatory context that links family and individual processes. Adolescents’ experiences of consistency or misalignment across home and school may shape whether digital regulation is interpreted as coherent, legitimate, and supportive of broader developmental goals (Eccles & Roeser, 2011; Sheridan et al., 2019). When school policies, classroom norms, and instructional practices align with autonomy-supportive parental mediation, adolescents may be more likely to experience digital regulation as understandable and purpose-driven rather than arbitrary or fragmented. In DSMDS, schools are therefore treated not merely as sites of rule enforcement, but as part of a broader regulatory ecology that can reinforce or complicate adolescents’ efforts to internalize digital self-regulation across contexts (Vazsonyi et al., 2017; Wang & Degol, 2016).

Schools may also function as interpreters of adolescents’ developmental signals, such as increasing self-management, emerging judgment, or ongoing struggles with distraction and peer pressure. Educators’ responses to these signals through graduated privileges, reflective conversations, collaborative norm-setting, or tighter external structure may shape whether students experience school-based regulation as supportive of competence and autonomy or as primarily controlling (Reeve, 2012). When schools coordinate with families around expectations, rationales, and developmental goals, regulatory support may become more coherent across contexts, potentially increasing the likelihood that adolescents can participate more actively in selecting, adapting, and maintaining regulatory supports in ways that fit their readiness and the demands of digital environments (Sheridan et al., 2019).

### Peer Context

Peers can be understood within DSMDS as a proximal co-regulatory context that shapes adolescents’ moment-to-moment digital self-regulation (Wang & Lee, 2026). During adolescence, digital engagement is often embedded in ongoing peer interaction, including group chats, shared content, social feedback, and norms around availability, making regulation not only an individual act but also a relational process (Brown & Larson, 2009; Nesi et al., 2018). Because peers are central to both relatedness need satisfaction and relatedness threat, they may either support or undermine adolescents’ internalization of digital regulation goals (Allen et al., 2012; Parent, 2023). When peer contexts reinforce norms that support balance, attentional focus, and school engagement, adolescents may experience regulation as socially endorsed and identity-consistent (Núñez-Regueiro & Wang, 2025; Ryan, 2001; Wentzel, 2017; Wentzel et al., 2014). Conversely, when peer expectations reward constant responsiveness, visibility, or participation, adolescents may experience disengagement as socially costly, increasing the likelihood that regulation is driven by external or introjected pressures rather than autonomous self-determination (Beyens et al., 2016; Gardner & Steinberg, 2005).

Peer influence in digital contexts is not reducible to norms alone. Rather, it often operates through co-regulatory dynamics involving reciprocity, shared expectations, reputational concerns, and coordinated routines (Wang & Lee, 2026). For example, adolescents may regulate use in concert with friends through mutually agreed-upon device-free periods, shared “Do Not Disturb” expectations during class, or collective boundaries around late-night messaging. Such practices may reduce the executive and motivational burden of self-regulation by distributing responsibility across the peer group and by making disengagement more socially legitimate (Veenstra et al., 2013). At the same time, peer dynamics may increase regulatory burden when belonging depends on rapid responsiveness, public posting, or participation in recurring digital social rituals. In SDT terms, these contingencies may heighten relatedness concerns and foster introjected pressures, making regulatory failure more likely even when adolescents possess relatively strong digital literacy competence (Beyens et al., 2016; Parent, 2023; Ryan & Deci, 2017).

Within DSMDS, peers may therefore be understood as a co-regulatory scaffold that shapes the salience of cues and rewards, the social meaning of regulation, and the feasibility of particular strategies. Peer contexts may support regulation when offline activities, shared expectations about availability, or group norms around device-free periods make disengagement more socially legitimate (Wang et al., 2019; Wentzel et al., 2014). Conversely, when adolescents anticipate relational penalties for being unreachable or less visible, peer contingencies may undermine those same efforts (Prinstein & Giletta, 2020). Framed this way, the peer context helps explain why digital regulation is often most difficult in socially evaluative settings and why intervention efforts may need to address not only individual skill-building, but also peer norms, group routines, and the social costs of disengagement (Dishion & Tipsord, 2011; Nesi et al., 2018).

### Policy Context

Within DSMDS, policy and platform design can be understood as distal regulatory conditions that shape how much self-regulatory burden is placed on adolescents, families, and schools. At this level, regulation is not enacted through moment-to-moment interpersonal guidance, but through broader rules, incentives, and design constraints that influence adolescents’ exposure to persuasive technologies, algorithmic reinforcement, and data practices (Livingstone & Third, 2017; Odgers & Jensen, 2020; Zuboff, 2019). Recent efforts to restrict youth access to social media and other high-engagement digital environments through age limits, institutional prohibitions, or platform-level controls reflect legitimate concerns about adolescent well-being (United Nations Educational, Scientific and Cultural Organization (UNESCO), 2023). DSMDS suggests that policies focused exclusively on restriction may not support the literacy, strategy use, and contextual conditions needed for longer-term self-regulation. From a developmental perspective, policy is most likely to be supportive when it not only limits harmful exposure, but also reduces manipulative design pressures and preserves opportunities for adolescents to develop literacy, judgment, and increasingly self-directed forms of digital regulation (Livingstone et al., 2018; Livingstone & Third, 2017; Valkenburg & Peter, 2013).

At the policy level, digital regulation may function as a higher-order scaffold that shapes the motivational and structural conditions under which adolescents attempt to self-regulate. Policies that focus narrowly on prohibition or age-based exclusion often assume that risk reduction follows primarily from reducing access. DSMDS raises a more developmentally qualified possibility: such approaches may sometimes reduce short-term exposure, but they may be less likely to support internalization when they are not paired with transparency, education, and conditions that make self-regulation more feasible across everyday contexts (Blum-Ross & Livingstone, 2018; Orben, 2020). More developmentally attuned policies may instead support digital self-regulation by requiring safer platform design, increasing transparency around persuasive features, promoting youth-centered protections, and encouraging educational opportunities that help adolescents understand and manage digital environments more intentionally (Livingstone et al., 2018; Livingstone & Third, 2017). For example, policy approaches might limit manipulative design features such as persistent notifications or opaque recommendation systems while still preserving developmentally appropriate opportunities for adolescents to practice judgment, boundary-setting, and increasingly self-directed digital use. In this regard, policy is not a substitute for family, school, or individual regulation, but part of the broader ecology that can either intensify or reduce the demands placed on adolescents’ developing self-regulatory capacities.

### Alignment Across Contexts

**Fig. 2:**
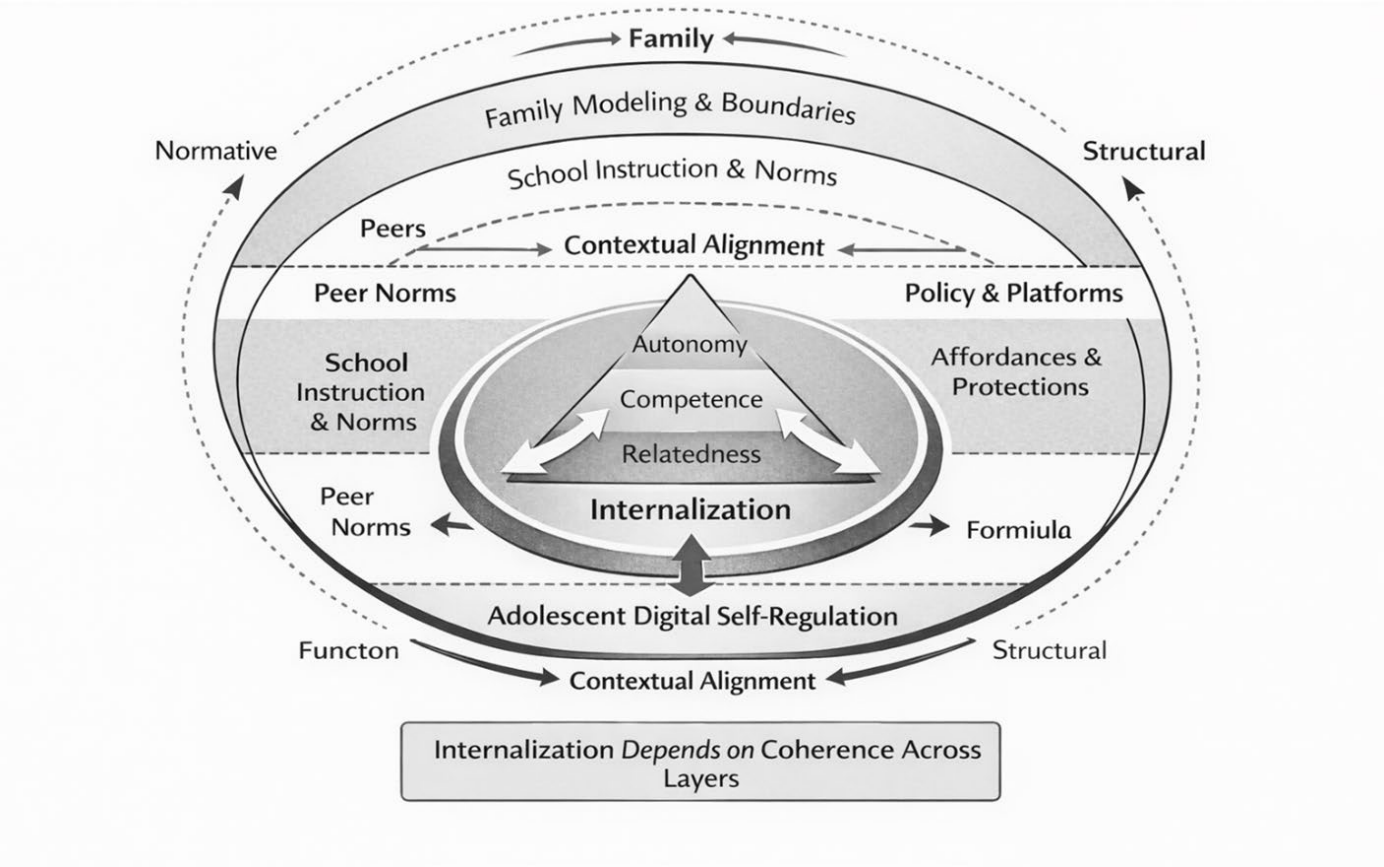
Normative and structural contexts of adolescent digital self-regulation

Effective support for adolescent digital self-regulation is likely to depend not only on what happens within families, schools, peer groups, or policy settings individually, but also on how coherently those contexts align. From the DSMDS perspective, adolescents are more likely to interpret digital regulation as legitimate, understandable, and developmentally supportive when expectations, rationales, and opportunities for practice are broadly consistent across ecological layers. For example, parental time-based boundaries may be more sustainable when schools reinforce attention-protective norms during the day and when peers do not impose strong social penalties for temporary disengagement. Likewise, school-based literacy instruction may be more effective when families use similar language around online judgment, self-monitoring, and boundary-setting, and when broader policy conditions reduce exposure to exploitative or highly persuasive platform features (Livingstone & Third, 2017; Reid Chassiakos et al., 2016). In this way, alignment across contexts is hypothesized to reduce regulatory burden, strengthen meaning-making, and increase the likelihood that adolescents experience digital regulation as a coherent developmental process rather than as a set of disconnected or competing demands (see Fig. 2 for a visual conceptualization).

### Implications for Practice, Policy, and Future Research

For practice, the DSMDS framework suggests that digital self-regulation should be cultivated through clear structure, explanation, collaboration, and developmentally calibrated participation in selecting and maintaining regulatory supports. Parents may support this process by collaboratively setting phone-free homework windows, explaining the rationale for those boundaries, and involving adolescents in adapting and maintaining those supports as their self-regulatory competence develops. Schools can reinforce similar processes by establishing structured device norms during instructional time, explicitly teaching attention management and digital literacy, and creating guided opportunities for students to reflect on when digital tools support versus undermine their learning (Hobbs, 2017; Organisation for Economic Co-operation and Development, 2021). Across contexts, the goal is to create conditions under which adolescents can understand why regulation matters and practice more self-endorsed forms of digital engagement, while recognizing that the effectiveness of such approaches requires further digital-specific testing.

At the policy level, the framework suggests that debates framed solely around banning versus permitting digital access are too narrow. Developmentally informed policies should attend to age, context, and purpose, recognizing that adolescents’ self-regulatory capacities are still developing and that external structure is most useful when it reduces burden without eliminating opportunities for skill-building (Blum-Ross & Livingstone, 2018; Orben, 2020). School policies that restrict access may be most developmentally supportive when paired with instruction that builds internal regulation skills. At broader levels, policies that limit manipulative design features, increase transparency around persuasive technologies, and support youth-centered protections may help reduce the self-regulatory demands placed on adolescents and the adults who support them (Livingstone et al., 2018; Livingstone & Third, 2017; United Nations Educational, Scientific and Cultural Organization (UNESCO), 2023).

The framework also points to several priorities for future intervention and program development. At present, there remains limited evidence on comprehensive, developmentally grounded programs explicitly designed to cultivate the digital literacy competencies and regulatory strategies described in DSMDS (Hobbs, 2017; Jones & Mitchell, 2016). Existing efforts are often fragmented, focused narrowly on risk avoidance, or detached from motivational and developmental theory. Advancing practice will require the intentional design and rigorous evaluation of programs that help adolescents build digital competence, critically understand online ecosystems, and practice multiple forms of self-regulation in developmentally responsive ways (Buchan et al., 2024; Jones & Mitchell, 2016). Such efforts may be especially valuable when integrated into existing social-emotional learning or academic curricula, where digital self-regulation can be treated as a core developmental competency alongside emotion regulation, goal-setting, and responsible decision-making (Organisation for Economic Co-operation and Development, 2021).

Beyond these practical implications, the framework also points to important theoretical and methodological priorities. Theoretically, future work should more precisely examine how motivational processes, executive capacities, and contextual scaffolds jointly shape the internalization of digital self-regulation across development. Methodologically, this will require designs that capture change over time and across settings, including longitudinal, transactional, and multi-level approaches that model reciprocal parent–adolescent, school–student, and peer processes. Intensive methods such as daily diary or ecological momentary assessment, especially when paired with family- and school-level data, may be especially useful for clarifying how regulatory strategies are learned, supported, and enacted in everyday life (Bolger & Laurenceau, 2013). Collectively, such work can move the field toward a more developmentally grounded and practice-relevant understanding of how adolescents learn to engage with digital environments in intentional, self-directed, and contextually supported ways.

### Limitations

Several limitations should be acknowledged. First, the empirical support for DSMDS is uneven across its components. Some elements of the model, such as the roles of executive function, autonomy-supportive scaffolding, and self-regulation more broadly, are well grounded in developmental and motivational theory, whereas other elements, particularly claims about the sustained use, sequencing, and internalization of digital regulation strategies across adolescence, remain less directly established in digital-specific empirical research. Accordingly, the framework should be interpreted as a synthesis of promising but uneven evidence, with several proposed relations still requiring direct empirical validation.

Second, the framework necessarily relies in part on theory and evidence developed outside explicitly digital contexts. Although this is appropriate for conceptual model building, it also means that some propositions are extrapolated from broader developmental, self-regulation, and motivational literatures rather than demonstrated specifically in adolescents’ digital lives. This is especially relevant for claims about developmental sequencing, internalization process, and the conditions under which external scaffolds are gradually transformed into more self-endorsed forms of regulation. These propositions are theoretically grounded, but they require further digital-specific testing.

Third, the model does not assume a single developmental pathway that applies uniformly across adolescents. The sequencing and effectiveness of digital self-regulation are likely to vary by age, developmental readiness, social context, platform features, and individual vulnerability, including differences in mental health, peer pressures, family resources, and exposure to highly persuasive or problematic digital environments. Future research will be needed to determine when the model applies most strongly, for whom, and under what ecological conditions.

Finally, DSMDS identifies developmental processes expected to operate across digital contexts, but it does not yet specify fully differentiated submodels for distinct activities such as gaming, messaging, entertainment streaming, social media, or AI-supported interaction. Future research should test whether the relative importance of literacy demands, motivational processes, peer pressures, persuasive design features, and regulatory strategies varies across these activity domains.

## Conclusion

This article introduces DSMDS as a developmentally grounded, self-determination-informed framework for understanding adolescents’ digital self-regulation as a coordinated system of literacy, strategy use, and contextual support. By distinguishing digital literacy competence as the knowledge and meaning-making foundation of regulation from regulatory strategies as the enactment layer through which that understanding is translated into action, the model reframes digital regulation as more than a question of access or control. It also specifies how parents, schools, peers, and policy contexts may function as scaffolds that are hypothesized to support or constrain adolescents’ opportunities to internalize intentional, self-directed forms of digital engagement.

The central implication of this framework is that effective digital regulation is a developmental process shaped by motivation, executive capacity, and contextual support. For adolescents, whose executive capacities are still maturing and whose digital lives are deeply embedded in social and institutional contexts, regulation is best understood as a contextually embedded process shaped by autonomy, competence, relatedness, and the structure of the environments in which digital engagement occurs. For educational psychology, this shifts the question from whether adolescents regulate digital behavior to how regulatory capacities are learned, scaffolded, and internalized over time. Framed this way, supporting adolescent digital well-being likely requires not only reducing risk, but also building and testing the capacities and contexts that may make intentional, developmentally adaptive digital engagement more feasible.
